# Effect of inactivated COVID-19 vaccine on the negative conversion of nucleic acid in asymptomatic or mild patients with COVID-19

**DOI:** 10.1186/s12879-023-08315-8

**Published:** 2023-06-30

**Authors:** Yifeng Luo, Qian Zhou, Xinyan Huang, Yuqi Ding, Xiangrong Ye, Jie Ding, Yukun Kuang, Yihao Liu, Sui Peng, Qingtang Zhu, Haibo Wang, Haipeng Xiao

**Affiliations:** 1 Division of Pulmonary and Critical Care Medicine, The First Affiliated Hospital of Sun Yat-sen University, Guangzhou, China; 2 Institute of Respiratory Diseases, Sun Yat-sen University, Guangzhou, China; 3 Clinical Trials Unit, The First Affiliated Hospital of Sun Yat-sen University, No. 58, Zhongshan Road 2, Guangzhou, 510080 Guangdong China; 4 Department of Gastroenterology and Hepatology, The First Affiliated Hospital of Sun Yat-sen University, Guangzhou, China; 5 Department of Liver Surgery, The First Affiliated Hospital of Sun Yat-sen University, Guangzhou, China; 6 Department of Endocrinology, The First Affiliated Hospital of Sun Yat-sen University, No. 58, Zhongshan Road 2, Guangzhou, 510080 Guangdong China; 7 Department of Microsurgery, Orthopaedic Trauma and Hand Surgery, The First Affiliated Hospital of Sun Yat-sen University, Guangzhou, China

**Keywords:** Negative conversion of nucleic acid, COVID-19, Inactivated vaccines, Booster vaccination

## Abstract

**Background:**

Negative conversion of nucleic acid was a key factor in deciding discharge or the end of isolation of asymptomatic or mild COVID-19 patients. We aimed to explore the effect of vaccination on the time to negative conversion after Omicron infection.

**Methods:**

This retrospective cohort study included asymptomatic or mild patients with COVID-19 admitted to Fangcang shelter Hospital from November 10, 2022 to December 2, 2022. The relationship between vaccination status and the time to negative conversion was analyzed by multiple linear regression.

**Results:**

A total of 2,104 asymptomatic or mild COVID-19 patients were included in the analysis, of whom 1,963 were vaccinated. The mean time to negative conversion of no vaccination, one dose, two doses, and three doses were 12.57 (5.05), 12.18 (3.46), 11.67 (4.86) and 11.22 (4.02) days, respectively (p = 0.002). Compared with no vaccination, two doses (β=-0.88, 95% CI: -1.74, -0.02, p = 0.045), and three doses (β=-1.51, 95% CI: -2.33, -0.70, p < 0.001) were both associated with shorter time to negative conversion. Comparing with two doses, booster dose was associated significantly with shorter time to negative conversion (β=-0.63, 95% CI: -1.07, -0.20, p = 0.004). Age was positively correlated with the time to negative conversion (β = 0.04, 95% CI: 0.02, 0.05, p < 0.001).

**Conclusion:**

Vaccination with inactivated vaccine and booster dose can shorten the time to negative conversion of asymptomatic or mild COVID-19 patients. The significant prolongation of time to negative conversion with increasing age suggests the promotion of vaccination, especially booster dose, particularly in the elderly.

## Introduction

The global pandemic of coronavirus disease-19 (COVID-19) has resulted in more than 600 million confirmed cases and 6.6 million deaths [1]. Since November 2021, the Omicron variant with the far surpassing ability in infection and immune escape than the Delta variant, has become the main epidemic strain in the world, which has led to a paradigm shift in the response to the pandemic [2, 3]. The large number of asymptomatic or mild patients with Omicron infection has result in serious social, economic and medical burden due to lock down, isolation, work stoppage and medical care.

The negative conversion of nucleic acid is a key factor in determining the discharge of patients and the end of isolation [4], therefore how to accelerate the time to negative conversion of nucleic acid has become a focus. It has been reported that age and disease conditions, including tumor, hypertension, diabetes, may affect the negative conversion [5–7]. Although COVID-19 vaccination is considered to reduce the infection, severity and mortality of Omicron [8–10], whether it can shorten the time to negative conversion of asymptomatic or mild patients with COVID-19 has not been reported.

Since October 2022, the Omicron variant has led to an epidemic in Guangzhou, Guangdong, China. This study used a large retrospective cohort of asymptomatic or mild patients with COVID-19 in Fangcang shelter Hospital to explore the impact of vaccination on the time to negative conversion of nucleic acids.

## Methods

### Participants

This study consecutively included asymptomatic or mild COVID-19 patients [4] admitted to the Fangcang shelter Hospital under the management of the First Affiliated Hospital of Sun Yat-sen University (FAH-SYSU) from November 10, 2022 to December 2, 2022 in Guangzhou. The inclusion criteria were: (1) Patients diagnosed as COVID-19 infection with positive nucleic acid test; (2) Patients diagnosed as asymptomatic or mild COVID-19 at admission without age limit. The exclusion criteria were: (1) Patients were still in hospital by December 2, 2022; (2) Patients admitted to hospital for more than once during the period of inclusion; (3) Patients without records of negative nucleic acid for two consecutive times after admission; (4) Patients who have received vaccines against SARS-COV-2 other than one of the five inactivated vaccines (Sinopharm Beijing Company, Sinopharm Wuhan Company, Sinovac, Shenzhen Biokangtai, and Institute of Medical Biology) [11]. This study was approved by the Ethics Committee of FAH-SYSU. Due to the nature of a retrospective study, the informed consent was waived.

### Clinical data collection and definition

In this study, we used the routinely collected data including age, gender, vaccination history, and nucleic acid test results and other medical history of patients. Vaccination history included vaccination status (whether vaccinated and the doses of vaccinations) and the corresponding dates. The standard dosing schedule for inactivated vaccines consisted of two doses administered at an interval of at least three weeks. The booster dose refers to the third dose of inactivated vaccines administered at an interval of at least 6 months after the second dose. Generally, in the Fangcang shelter Hospital, patients may receive daily nucleic acid test within five days since being positive. Negative conversion of nucleic acid test was defined that patients had two consecutive negative results in two separate days. Time to negative conversion of nucleic acid was defined as the time from being positive to the second test of two consecutive negative tests.

### Statistical analysis

Continuous variable with normal distribution was described using mean and standard deviation (SD) compared using t test or ANOVA for two or more than two groups. Nonnormal distributed data was described using median and interquartile range (IQR) and compared by Wilcoxon rank-sum test or Kruskal Wallis test. Categorical variable was presented as frequency and proportion and compared via Chi-square test. Multiple linear regression model was performed to evaluate the association of doses of vaccination and time to negative conversion of nuclear acid adjusting available potential confounders including age, gender and and medical history of patients. The statistical analyses were performed with SAS 9.4 and R 4.1.2. With 2-sided testing, P < 0.05 was considered statistically significant.

## Results

### Baseline characteristics

From November 10 to December 2, 2022, a total of 2104 asymptomatic or mild patients with COVID-19 were included. Among them, 119 (5.7%), 39 (1.9%), 540 (25.7%), and 1406 (66.8%) patients did not received vaccine or received 1, 2, and 3 doses of vaccine, respectively. Table 1 presents the baseline characteristics of all patients in this study. Among them, 1073 were males (51.0%). The median age of the patients was 38.00 years old. The median time from the last vaccine injection to this admission was 327.00 days (IQR: 280.00, 399.00). There were significant differences (p < 0.001) in the baseline characteristics between the groups among patients who were not vaccinated and those who received 1, 2, and 3 doses of vaccine. The mean time to negative conversion of no vaccination, one dose, two doses, and three doses were 12.57 (5.05), 12.18 (3.46), 11.67 (4.86) and 11.22 (4.02) days, respectively (p = 0.002).

**Table 1:** Baseline Characteristics

	level	Overall	Not vaccinated	Dose 1	Dose 2	Dose 3	p
Number		2, 104	119 (5.7%)	39 (1.9%)	540 (25.7%)	1, 406 (66.8%)	
Gender (%)	Male	1,073 (51.0)	83 (69.7)	24 (61.5)	282 (52.2)	684 (48.6)	< 0.001
	Female	1,031 (49.0)	36 (30.3)	15 (38.5)	258 (47.8)	722 (51.4)	
Age (median [IQR])		38.00 [31.00, 49.00]	36.00 [27.50, 47.00]	34.00 [27.00, 45.00]	34.50 [28.00, 46.00]	39.00 [33.00, 49.00]	< 0.001
Age (%)	< 18y	96 (4.6)	21 (17.6)	6 (15.4)	69 (12.8)	0 (0.0)	< 0.001
	18-60y	1,984 (94.3)	93 (78.2)	33 (84.6)	465 (86.1)	1,393 (99.1)	
	> 60y	24 (1.1)	5 (4.2)	0 (0.0)	6 (1.1)	13 (0.9)	
Chronic Disease History (%)	yes	1,812 (86.1)	89 (74.8)	33 (84.6)	477 (88.3)	1,213 (86.3)	0.002
	no	292 (13.9)	30 (25.2)	6 (15.4)	63 (11.7)	193 (13.7)	
Surgical history(%)	yes	1,787 (84.9)	96 (80.7)	30 (76.9)	462 (85.6)	1,199 (85.3)	0.268
	no	317 (15.1)	23 (19.3)	9 (23.1)	78 (14.4)	207 (14.7)	
Allergic history(%)	yes	1,980 (94.1)	108 (90.8)	36 (92.3)	512 (94.8)	1,324 (94.2)	0.371
	no	124 (5.9)	11 (9.2)	3 (7.7)	28 (5.2)	82 (5.8)	
Last vaccine to being positive (median [IQR])		327.00 [281.00, 406.00]	NA	476.00 [361.50, 498.50]	465.00 [441.50, 489.00]	307.00 [268.00, 334.00]	< 0.001
Time to negative conversion (median [IQR])		11.00 [9.00, 13.00]	12.00 [10.00, 15.00]	12.00 [10.00, 15.00]	11.00 [9.00, 14.00]	11.00 [9.00, 13.00]	0.001
Time to negative conversion (mean [SD])		11.43 (4.31)	12.57 (5.05)	12.18 (3.46)	11.67 (4.86)	11.22 (4.02)	0.002

IQR: interquartile range; SD: standard deviation

### Effect of vaccination status on time to negative conversion of nucleic acid

We first performed linear regression analysis on all 2104 patients to evaluate the association of vaccination stutus with time to negative conversion of nucleic acid. Multiple linear regression showed that compared with no vaccination, two doses (β=-0.88, 95%CI: -1.74, -0.02, p = 0.045), and three doses (β=-1.51, 95%CI: -2.33, -0.70, p < 0.001) significantly shortened the time to negative conversion. The average time to negative conversion of nucleic acid of two doses and three doses reduced 0.88 and 1.51 days comparied with that of no vaccination, respectively. In addition, comparing with two doses, three doses can significantly shorten the time to negative conversion (β=-0.63, 95%CI: -1.07, -0.20, p = 0.004) (Table 2). The average time to negative conversion of nucleic acid of three doses reduced 0.63 days comparied with that of two doses.We also found that age was positive correlated with the time to negative conversion (β = 0.04, 95% CI: 0.02, 0.05, p < 0.001) (Table 2). When the age increased by one year, the average time to negative increased by 0.04 days. We also estimated that when the age increased by ten years, the average time to negative increased by 0.36 days (β = 0.36, 95% CI: 0.21, 0.52, p < 0.001).

**Table 2:** Multiple linear regression between vaccination status and time to negative conversion in all included patients

Factor	Level	Coefficient [95% CI]	p
Vaccination	Dose 1 vs. 0	-0.35 [-1.90, 1.20]	0.657
	Dose 2 vs. 0	-0.88 [-1.74, -0.02]	0.045
	Dose 3 vs. 0	-1.51 [-2.33, -0.70]	< 0.001
	Dose 2 vs. 1	-0.53 [-1.92, 0.87]	0.459
	Dose 3 vs. 2	-0.63 [-1.07, -0.20]	0.004
	Dose 3 vs. 1	-1.16 [-2.53, 0.21]	0.097
	Dose 3 vs. No Booster	-0.81 [-1.21, -0.41]	< 0.001
Age (per year)	~	0.04 [0.02, 0.05]	< 0.001
Gender	Female vs. Male	-0.18 [-0.56, 0.19]	0.332
Chronic Disease history	~	0.15 [-0.40, 0.70]	0.597
Surgical history	~	-0.10 [-0.63, 0.43]	0.707
Allergic history	~	0.02 [-0.77, 0.80]	0.969

CI: confidence interval

Then, regression analysis was carried out among 1963 vaccinated patients, adding the factor of the time from the last vaccine to being positive. Multiple linear regression showed that the booster vaccination could significantly shorten the time to negative conversion. Three doses versus one dose (β=-1.96, 95%CI: -3.37, -0.54, p = 0.007), three doses versus two doses (β=-1.52, 95%CI: -2.15, -0.89, p < 0.001), both can significantly shorten the time to negative conversion. The average time to negative conversion of nucleic acid of two doses and three doses reduced 1.96 and 1.52 days comparied with that of one dose, respectively. Interestingly, the longer the time from the last vaccination to admission, the shorter the time to negative conversion, though the regression coefficient was small (β=-0.01, 95% CI: -0.01, -0.00, p < 0.001) (Table 3). When the the time from the last vaccination to admission increased by one day, the average the time to negative conversion reduced 0.01 day. When the time from the last vaccination to admission increased by one month, the average the time to negative conversion would decrese to 0.18 day (β= -0.18, 95% CI: -0.27, -0.08, p < 0.001).

**Table 3:** Multiple linear regression between vaccination status and time to negative conversion in all vaccinated patients

Factor	Level	Coefficient [95% CI]	p
Vaccination	Dose 2 vs. 1	-0.44 [-1.82, 0.94]	0.532
	Dose 3 vs. 1	-1.96 [-3.37, -0.54]	0.007
	Dose 3 vs. 2	-1.52 [-2.15, -0.89]	< 0.001
	Dose 3 vs. No Booster	-1.55 [-2.17, -0.93]	< 0.001
Age (per year)	~	0.05 [0.03, 0.06]	< 0.001
Last vaccine to admission by day	~	-0.01 [-0.01, -0.00]	< 0.001
Gender	Female vs. Male	-0.20 [-0.58, 0.18]	0.314
Chronic Disease history	~	-0.07 [-0.64, 0.50]	0.82
Surgical history	~	-0.01 [-0.54, 0.53]	0.984
Allergic history	~	0.21 [-0.60, 1.02]	0.615

CI: confidence interval

## Discussion

Using the large retrospective cohort of asymptomatic or mild COVID-19 patients admitted to Fangcang shelter Hospital during the Omicron epidemic, this study analyzed the relationship between the vaccination status of the inactivated vaccine against SARS-CoV-2 and the time to negative conversion of nucleic acid. We found that inactivated COVID-19 vaccine and booster dose may shorten the time to negative conversion among asymptomatic or mild patients with COVID-19.

These results are comparable to those of other studies on vaccines. According to a previous publication, compared with non-vaccination, the protection rates of inactivated vaccines in preventing Omicron-infected patients from progressing to severe or fatal disease reached 88.6% and 91.7%, respectively [8]. In addition, booster vaccination can significantly improve the Omicron serum antibody titer and the cellular immunity induced by antigen-specific CD8 + T cells [12, 13], therefore further reduce the risk of severity, mortality and disease progression [8, 14, 15].

The predominant virus strain in Guangzhou is Omicron BA.5 [16], which has a relatively low pathogenicity but more transmissibility than the other COVID-19 variant. It requires us to investigate the role of vaccines in another aspect, whether it can shorten the hospitalization or isolation time of asymptomatic or mild patients to reduce the economic and medical burden. Our study found that vaccination, especially booster dose can significantly shorten the time to negative conversion, suggesting that the inactivated vaccines can accelerate the recovery of Omicron infection.

In addition, we also found that there is a positive correlation between age and the time to negative conversion, which is consistent with previous reports [6]. In China, around 25 million persons over the age of 60 have not been immunized, and about 82 million have not received booster dose [17]. During the Omicron epidemic in Hong Kong in early 2022, the risk of COVID-19-associated death of unvaccinated patients > 60 years old was 21.3 times that of elderly who received 2–3 doses of vaccines [18], and the protection rate of booster for preventing the elderly from developing severe or fatal disease was more than 95% [19]. This study serves as a reminder that promoting vaccination and booster dose among the elderly is an essential way for combating the Omicron epidemic.

The major limitation of this study is the retrospective nature. Though we have collected and included all possible clinical information, the available information of this study may still be limited and other confounding factors may exist. In addition, the history of previous COVID-19 infection and the specific type of infecting subvariants were not available.

## Conclusions

Inactivated COVID-19 vaccine and booster dose may shorten the time to negative conversion among asymptomatic or mild patients with COVID-19. In addition, we also found that the older the patient, the longer time to the negative conversion. Therefore, efforts are needed be paid to improve the rate of vaccination and booster dose, especially in the elderly, which would help reduce the burden of the disease on our healthcare system and control the spread of COVID-19.

## Data Availability

The datasets analysed during the current study are available from the corresponding author on reasonable request.

## Abbreviations

COVID-19: Coronavirus disease-19
SD: Standard deviation
IQR: Interquartile range
